# Peroxisome Proliferator-Activated Receptor and Age-Related Macular Degeneration

**DOI:** 10.1155/2008/389507

**Published:** 2008-01-27

**Authors:** Alexandra A. Herzlich, Jingsheng Tuo, Chi-Chao Chan

**Affiliations:** Laboratory of Immunology, National Eye Institute, National Institutes of Health, Bethesda, MD 20892-1857, USA

## Abstract

Age-related macular degeneration (AMD) is the leading cause of new blindness in the western world and is becoming more of a socio-medical problem as the proportion of the aged population increases. There are multiple efforts underway to better understand this disease process. AMD involves the abnormal retinal pigment epithelium (RPE), drusen formation, photoreceptor atrophy, and choroidal neovascularization. Peroxisome proliferator-activated receptors (PPARs) play an important role in lipid degeneration, immune regulation, regulation of reactive oxygen species (ROSs), as well as regulation of vascular endothelial growth factor (VEGF), matrix metalloproteinase-9 (MMP-9), and docosahexaenoic acid (DHA). These molecules have all been implicated in the pathogenesis of AMD. In addition, PPAR gamma is expressed in RPE, an essential cell in photoreceptor regeneration and vision maintenance. This review summarizes the interactions between PPAR, AMD-related molecules, and AMD-related disease processes.

## 1. INTRODUCTION

Improvements in public health and medical advancements have led to increasing lifespan among the population today and consequently, a mounting burden of many disorders of deteriorating body systems such as age-related macular degeneration (AMD). Currently AMD is the leading cause of blindness in developed countries [1]. With the general aging of the population, this debilitating disease promises to become an even bigger health care problem. As the demand for therapy increases, much effort is being directed toward the elucidation of the mechanisms underlying AMD pathogenesis.

Peroxisome proliferator-activated receptors (PPARs) are members of the steroid/thyroid nuclear receptor superfamily of ligand-activated transcription factors. PPARs are involved in lipid and glucidic metabolism, immune regulation, and cell differentiation. Because of these functions, PPARs and their synthetic agonists have been marketed as fibrates and thiazolidinediones for hypercholesterolemia and type 2 diabetes mellitus, respectively [2]. There is much speculation regarding the potential role of PPARs in other disease mechanisms. Recently, PPARs have been associated with age-related changes in Alzheimer’s disease [3] and Parkinson’s disease [4], suggesting that PPARs might also play a role in the pathogenesis of AMD.

## 2. AGE-RELATED MACULAR DEGENERATION

The normal aging process of the eye can include a spectrum of changes in the eyes [5] as follows. Photoreceptors decrease in density, retinal pigment epithelium (RPE) undergoes loss of melanin; formation of lipofuscin granules, and accumulation of residual bodies; and basal laminar deposits accumulate in Bruch’s membrane. AMD is a degenerative disease of the central portion of the retina (the macula) which results primarily in loss of central vision [6]. The disease can progress in two different ways and, therefore, can be classified into a dry form (geographic atrophy) and a wet form (neovascular AMD).

In both subtypes of AMD, the RPE is a crucial cell in the pathogenesis of AMD [6]. A pivotal function of the RPE is the phagocytosis of the outer segments of the photoreceptors and subsequent regeneration of the rods and cones. As one ages, metabolic waste builds up and imposes an increasing burden on the RPE. The waste, now partially degraded in a phagolysosome, is visualized histologically as residual bodies and serves as a substrate for lipofuscin formation. These residual bodies increase in number until they are extruded and accrue in Bruch’s membrane, thickening the membrane itself and forming dome shaped basal linear deposits in Bruch’s membrane referred to as drusen. When the deposits become large (> 125 *μ*m in diameter), soft (amorphous and poorly demarcated), and confluent, they cause interruptions in the choroidal capillaries, compromising blood flow within the RPE layer. The extracellular deposits in Bruch’s membrane also instigate chronic inflammation, promoting invasion by phagocytes and other immune cells, cytokine release, and production of reactive oxygen species (ROSs) [7].

The retina, because of its high oxygen consumption, its high levels of cumulative irradiation, and its composition of polyunsaturated fatty acids, which are readily oxidized and can initiate a cytotoxic chain reaction, is an ideal environment for the generation of ROS [8]. Moreover, the process by which RPE phagocytizes is itself an oxidative stress that results in ROS generation. The combined effects from chronic sustained inflammation and ROS generation promote the development of RPE damage seen in AMD [6, 9, 10]. Thinning or destruction of theRPE leads to its degeneration and to the subsequent death of rods and cones that depend on the RPE for their nutrition. This translates into visual loss. As the RPE degenerates, choriocapillaris beneath the RPE becomes less fenestrated, reducing the transport of macromolecules between the retina and choroidal blood supply and then disappearing altogether, creating a hypoxic environment. Hypoxia then increases the secretion of growth factors such as vascular endothelial growth factor (VEGF) that promotes choroidal neovascularization (CNV). The friable, small vessels comprising CNV are easily damaged and leak, creating the wet or exudative form of macular degeneration. The other more-common and less-severe form, termed dry AMD, occurs in the absence of neovascularization and with a region of atrophy in a geographic distribution [6].

### 2.1. Risk factors for AMD

The etiology of AMD remains elusive. A major feature of AMD is its association with age, with the highest prevalence among those 85 years of age or older [1]. Other certain risk factors include smoking and family history or genetics [6, 11–17]. There have been recent studies showing certain association between AMD and *CFH* [18–23], *LOC38775/ARMS2* (age-related maculopathy susceptibility 2) [24–27], *HrtA-1* [28, 29], and *APOE* [30–34]genes. Recently, VEGF single nucleotide polymorphism and matrix metalloproteinases (MMP)-9 microsatellite polymorphism are reported to be associated with wet AMD [35–37]. Studies have also considered an association between exposure to sunlight and AMD [6].

The Age-Related Eye Disease Study (AREDS), a controlled randomized clinical trial reports the use of high doses of antioxidants (vitamin C, vitamin E, and beta carotene) and zinc reduce the risk of advanced AMD by about 25% in patients with moderate risk of developing AMD [38]. Supplementation of various nutrients in the literature have demonstrated risk reduction for AMD, and these findings support the potential role of PPARs in AMD, especially since diet is an important modifiable risk factor when discussing PPARs, which regulate lipid metabolism and homeostasis [39, 40]. PPAR is one of the two characterized types of polyunsaturated fatty acid-responsive transcriptional factors. Because humans do not have the capability for de novo synthesis of essential fatty acids, which are particularly rich in long-chain polyunsaturated fatty acid (LCPUFA), we are dependant on dietary sources of these compounds [9]. Importantly, a recent AREDS study has demonstrated that participants reporting high-dietary intake of lutein/zeaxanthin, an LCPUFA which counteracts photochemical damage and generation of reactive oxygen species that attack cellular lipids, proteins, and other nuclear material, are statistically less likely to have advanced AMD (both neovascularizationand geographic atrophy) or large or extensive intermediate drusen than thosereporting lowest dietary intake of lutein/zeaxanthin [41]. Thus, it is possible that the beneficial effects of lutein/zeaxanthin LCPUFAs are related to their ability to activate fatty acid-responsive PPARs, suggesting a protective role of PPARs in AMD pathogenesis.

### 2.2. Clinical presentation

Though the etiology of AMD remains unclear, the clinical progression of this disease is well characterized. With dry AMD, patients may complain of a gradual loss of vision, from several months to years, in one or both eyes due to progressive loss of photoreceptors [42]. This gradual loss of vision is often first noticed as difficulty in reading or driving, scotomas, or increased reliance on brighter light or a magnifying lens for tasks that require fine visual acuity [43]. Vision loss that has occurred acutely over a period of days or weeks may represent wet AMD due to subretinal/retinal hemorrhage resulting from leakage or breaks of choroidal neovascular vessels. These patients may report an acute distortion in vision due to retinal hemorrhage, especially distortion of straight lines, or loss of central vision. Symptoms of wet AMD usually appear in one eye although AMD pathology is generally present in both eyes [44].

### 2.3. Pathological findings

The nonneovascular abnormalities in AMD include drusen as well as abnormalities of the RPE highlighted by accumulation of lipofuscin granules. The main component of lipofuscin is A2E, which is cytotoxic to RPE and induces RPE apoptosis. Clinically, drusen are round, dull yellow lesions, located under the sensory neuroretina and RPE, which upon fluorescein angiography, light up and stain late with no leakage. Histologically this material corresponds to the abnormal thickening of the inner aspect of Bruch’s membrane. The thickening involves basal laminar deposits, collagen accumulation between the plasma membrane of the RPE cells and the inner aspect of the basement membrane of the RPE, as well as basal linear deposits outside the RPE basement membrane referred to as drusen [6].

How and why drusen develop is unknown, however much is deduced from its contents. Drusen often have a core of glycoproteins and their outer domes contain crystallins, chaperone proteins, apolipoprotein E, vitronectin, proteins related to inflammation (amyloid P, C5, and C5b-9), and sometimes fragments of RPE cells [45]. Drusen appear as electron-dense granules within the inner aspect of Bruch’s membrane. The thickening of the membrane causes a sharp reduction in fluid and nutrient transport across the membrane. Its diminished function also results in decreased cell adhesion and anoikis of the photoreceptors, RPE cells, and possibly choriocapillaris endothelial cells [6]. These deposits around Bruch’s membrane are also the cause of chronic local inflammation further promoting AMD development and progression.

The presence of drusen may lead to RPE degeneration and subsequently, deterioration of photoreceptors, which are dependent upon maintenance by RPE [46]. When the atrophy of the RPE and photoreceptors covers a distinct and contiguous area, it is termed geographic atrophy. Histologically, geographic atrophy is characterized by roughly oval patches of hypopigmentation as a consequence of RPE atrophy. The underlying choroidal vessels are more readily visible and the outer retina may appear thin secondary to loss of the photoreceptor and RPE cells. At the periphery of the hypopigmented regions there may be hyperpigmented changes from RPE cell proliferation. If the atrophy is less defined, with a mottled appearance, then it is called nongeographic atrophy. If the disease continues to progress, there comes a point when the components of the drusen begin to disappear; this is termed regressed drusen [46]. Additionally there may be small pinpoint glistening of the drusen where calcium has been deposited.

The third key component of AMD is choroidal neovascularization [47]. With the thinning and destruction of the RPE the underlying choriocapillaries become less fenestrated, impairing transport of macromolecules, such as oxygen, between the retina and choroidal blood supply. The resulting hypoxia stimulates neovascularization through vascular endothelial growth factor (VEGF). VEGF, which will be discussed in more detail below, acts as a stimulus for neovascularization [48]. There can be both new vascular growths from the choroidal vessels, growing through Bruch’s membrane into the subretinal space. Clinically CNV appears as a purple-grey discoloration beneath the retina. With the increase in blood flow within the retina due to CNV, there may even be a focal sensory retinal detachment and cystoid edema. New vessels also promote fibroblast proliferation and disruption of normal retinal architecture. Moreover, these neovascular blood vessels are extremely leaky, and hemorrhage from these friable vessels leads to sudden vision loss secondary to accumulation of fluid or blood in the subretinal space and/or within the retina itself [49].

## 3. PEROXISOME PROLIFERATOR-ACTIVATED RECEPTORS

**Figure 1:**
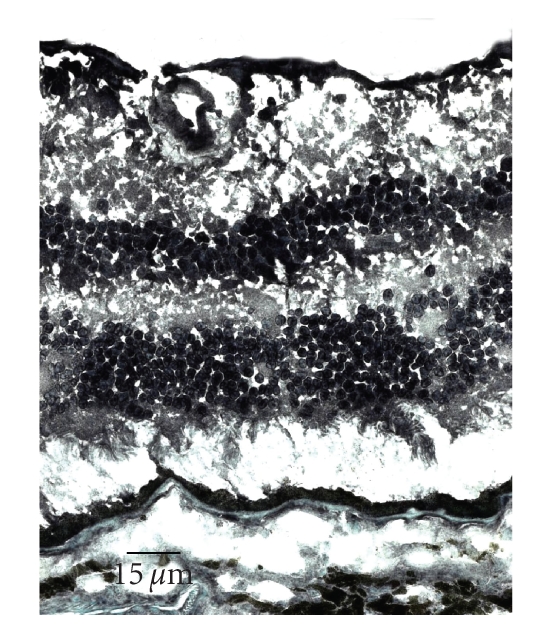
Microphotograph showing normal human retina stained for PPAR*γ* in the ganglion cell, inner nuclear layer, outer nuclear layer, and RPE (avidin-biotin-complex immunoperoxidase).

*Peroxisome proliferator-activated receptors* (PPARs) seem to be associated with chronic diseases such as diabetes mellitus, obesity, atherosclerosis, cancer, and neurodegenerative diseases [2, 4, 50]. Like androgens, steroids, retinoid, and thyroid hormone receptors groups, PPARs are members of nuclear receptor superfamily of ligand-activated transcription factors [2]. Though they are among the best-categorized nuclear receptor families, the evolution of these molecules remains unclear. PPARs have three known subtypes: *α*, *β*, and *γ*. The *α* subtype is present in adipose tissue, liver, brain, heart, and skeletal muscle. A synthetic agonist to this subtype has been created as a cholesterol-lowering therapy. The PPAR *β* subtype, also known as *δ* or NUC1, is present in the gut, kidney, brain, and heart. PPAR*γ*, the subtype most widely studied, is expressed on adipocytes, colon, brain, renal epithelium, monocytes, and macrophages. The *γ* subtype is the model for therapy such as thiazolidinediones (troglitazone, rosiglitazone, pioglitazone) for increased insulin sensitivity in noninsulin-dependent diabetes (type 2) [51, 52]. This receptor is also expressed in the retina, specifically in the RPE and choroidal vascular endothelial cells [53]. Figure 1 shows positive immunoreactivity against PPAR*γ* in the normal human retina. The association of PPAR with RPE cells, as well as neuronal cells, supports the hypothesis that PPAR may play a role in the pathogenesis of AMD; therefore, PPAR may present a possible target for AMD treatment.

In response to binding by fatty acids, PPARs form heterodimers with retinoid X receptor (RXR), and the PPAR-RXR heterodimer binds to specific response elements (PPREs) consisting of a direct repeat of the nuclear receptor hexameric DNA core recognition motif spaced by one nucleotide to influence the transcription of numerous target genes [54]. Because PPAR is widely expressed as a transcription factor, it also plays a role in many processes including lipid homeostasis, glucose regulation, inflammation, atherosclerosis, ischemia, cancer, and neurodegenerative diseases [2, 36, 37, 54–62] with the subtypes overlapping in activity, function, and location.

## 4. PROPOSED MECHANISMS OF AMD AND THE LINKS TO PPAR

The etiology of AMD is not well understood, an explanation in itself for the various proposed mechanisms for how and why AMD progresses. Theories include aging, oxidative stress, endoplasmic reticulum stress, and inflammation. Interestingly, these processes are shared among diseases with similar pathophysiological changes to those seen in AMD and also involve PPAR.

Oxidative stress arises from a significant increase in reactive oxygen species (ROS) concentration and/or a decrease in detoxification mechanisms. ROS include free radicals, hydrogen peroxide, and singlet oxygen. There are many natural sources of oxidative stress such as exposure to environmental oxidants, ionizing and UV radiation, heat shock, and inflammation. The ROSs usually have one or more unpaired electrons in their outer orbits, and in order to achieve a stable state, extract electrons from other molecules, which themselves become unstable, causing a chain reaction [8]. High levels of oxidative stress exert a toxic effect on biomolecules, such as DNA, proteins, and lipids. As we know ROS may start an oxidative cascade, mediated in part by ROS-induced activation of NF-*κ*B, STAT, and AP-1transcription factors, altering the composition of the cellular membrane, changing protein conformations, and lead to an upregulation of proinflammatory genes and cytokines, further potentiating damage [62, 63].

Oxidative stress plays a role in ischemic-reperfusion injuries, atherosclerosis, hypertension, inflammation, cystic fibrosis, type 2 diabetes, Alzheimer’s, and Parkinson’s disease [62]. Oxidative stress has also been linked to aging [64]. The retina has a very high concentration of lipids [9] and therefore easily falls pray to such mechanisms of destruction [8].

Oxidative stress such as aging and light exposure is considered to be associated with AMD. RPE and photoreceptors are particularly susceptible to oxidative stress because of high oxygen consumption by photoreceptors [8], high concentration of LCPUFA in the outer segments [65], exposure to visible light, and presence of lipofuscin, a photo-inducible generator of ROS in RPE [66, 67]. Clinical data supporting a beneficial effect of antioxidants in AMD provide direct validation of the role of oxidative injury in AMD treatment. Subgroup analysis of a multicenter, randomized, placebo-controlled AREDS trial revealed that an antioxidant cocktail of vitamins C and E, *β*-carotene, and zinc can reduce the progression of moderate atrophic AMD to late-stage disease [38]. Epidemiologic data showing that smoking leads to a significantly increased risk of the disease is consistent with the antioxidant approach as smoking is known to depress antioxidants such as vitamin C and carotenoids, and to induce hypoxia and ROS generation [68, 69].

PPARs are known to stimulate peroxisome enlargement and proliferation, as well as upregulation of *β*-oxidation enzymes. Since the peroxisome houses a variety of oxidative metabolic processes, they are an obvious cause of oxidative stress [64]. Oxidative damage and proinflammatory cytokines, TNF-*α*, INF-*γ*, and MMPs have been cited to play roles in each of the disease processes mentioned above [3, 50, 70–74], establishing PPAR as a common link between them.

Another theory regarding drusen formation involves a phenomenon known as endoplasmic reticulum (ER) stress. The ER is central to protein and lipid synthesis and maturation, as most newly formed proteins are assembled in the ER. Incorrectly folded proteins tend to form aggregates that are harmful to the cells and thus, ER-resident and/or visiting chaperone molecules facilitate protein folding and clearance of terminally misfolded proteins [75]. Any condition which impairs protein folding, for example, mutations in proteins that affect folding or ER malfunction, is termed ER-stress. Increased ER-stress, therefore, leads to protein and lipid buildup within cells, and this buildup in the eye might translate into RPE damage and drusen deposition.

The argument for a role for ER stress in AMD pathogenesis is supported by the well-characterized role of ER stress in several AMD-related neurodegenerative diseases. Alzheimer’s disease and Lewy Body diseases, such as Parkinson’s disease, are characterized by deposition of abnormal substances, which may parallel the abnormal deposition of drusen in the eye. The classical histopathological hallmarks of Alzheimer’s disease [3, 4] include deposition of fibrillar amyloid in neuritic plaques as well as intracellular deposits of hyperphosphorylated tau protein. This results in the formationof neurofibrillary tangles and finally neuronal death, causing progressive memory loss and decline in cognitive functions [4]. In Parkinson’s disease, suffering dopaminergic neurons are found to contain Lewy bodies and neuromelanin, an end product in catabolism by autoxidation [3]. In atherosclerosis there are abnormal lipid depositions in blood vessels leading to plaque formation and partial occlusion of these vessels [76]. In an AMD model of *Ccl2* ^−/−^/*Cx3cr1^−/−^* deficient mice abnormal ER protein is detected and associated with disease pathogenesis [75].

Recent articles have discovered a role for PPAR in ER stress. Dirkx et al. found that absence of peroxisomes in hepatocytes had repercussions on different subcellular compartments, including mitochondria, ER, and lysosomes [77]. Another study found that intracellular calcium mobilization by PPAR*γ* ligands in rat liver epithelial cells interferes with proper protein foldingin the ER, thus promoting ER stress [73]. A third article discovered that under conditions of impaired translation, PPAR*γ* ligands stimulate the expression of a number of ER stress-responsive genes, such as GADD 153, BiP, and HSP70 in rat pancreatic *β* cells. They concluded that PPAR*γ* ligands induce ER stress [78].

In addition to the obvious parallels, between amyloid, Lewy bodies, cholesterol, and drusen, there are also similar processes such as inflammation that may play a role in inciting the damage associated with each disease.

Various immunological molecules and inflammatory mediators, cytokines, and chemokines have been identified in AMD lesions [79, 80]. Many of them are produced locally by RPE, choroid, and retina [81]. It has been hypothesized that RPE dysfunction is the critical event in drusen formation, making drusen a product of a localized inflammatory response, possibly involving HLA antigens and the complement system [82]. The hypothesis is based on many different findings scattered among the literature and within different fields of medicine. Drusen, the hallmark of AMD, are found higher in membranoproliferative glomerulonephritis II (MPGNII), a complement-medicated immune deficiency. These cuticular drusen are identical, clinically, histologically, and immunohistochemically to the drusen in AMD [83–85]. Drusen has also been cited as having similar features to lipid-laden plaques of atherosclerosis [82, 86]. The relationship here is inferred from the histological as well as local inflammatory similarities between dysfunctional endothelial cells and the subendothelial deposition of modified LDL-cholesterol in atherosclerotic deposits within arterial vessels to those of drusen in the eye [86–88]. In addition, molecules such as MMP-9 seem to be involved in both processes. Inhibition of MMP-9 in atherosclerotic lesions has been cited to oppose remodeling, as suggested by the inhibition of intimal thickening and outward arterial remodeling [89]; while in AMD it is thought to be involved in microvessel formation duringearly phases of angiogenesis, in the reabsorption ofneovascularization, and in involution and regression of vessels inlater stages [90]. Similarities to the local inflammatory components seen in Alzheimer’s also support this theory where accumulations of neurofibrillary tangles or insoluble deposits of beta amyloid peptide are the inciting agents of local inflammation [86].

The association between *complement factor H (CHF)* single nucleotide polymorphisms and increased risk of AMD also uncovers an important link between the complement system (inflammation) and the development of maculopathy (AMD) [18–20, 91]. The gene for *CHF* is located within the chromosomal region (1q32) linked to AMD [82, 92]. The *CHF* gene encodes a protein, complement response factor (CRF), that functions as part of the complement system and has been found in drusen from AMD patients [82, 93]. Furthermore, the same environmental risk factors, smoking, that influence levels of complement in serum are also associated with increased risk of developing AMD [86, 94].

In Alzheimer’s, atherosclerosis, and AMD similar local proinflammatory pathways are stimulated, thereby leading to the deposition of activated complement components, acute-phase proteins, and other inflammatory mediators in tissues affected by each disease process. The cumulative impact is chronic tissue-specific low-grade inflammation exacerbating the effects of the primary pathogenic lesion [86]. PPARs act to inhibit many proinflammatory genes, which may result in protection of these diseases.

## 5. MOLECULES THAT INTERACT WITH PPAR AND THEIR RELATIONSHIP WITH AMD: AN INTRODUCTION TO VEGF, MMP, AND DHA

### 5.1. Vascular endothelial growth factor A, VEGF

VEGF was first identified in the early 1970s as a tumor-angiogenesis factor that is mitogenic to capillary endothelial cells in human tumors [95]. VEGF is now recognized as an essential regulator of normal and abnormal vessel growth. It regulates both vascular proliferation, as well as permeability, and functions as an antiapoptotic factor for newly formed blood vessels [95]. VEGF is expressed in response to hypoxia, oncogenes, or cytokines [96]. In this process, VEGF binds to and stimulates autophosphorylation of two distinct receptor tyrosine kinases, VEGFR1 or Flt-1 (fms-related tyrosine kinase 1) and VEGFR2 or KDR/FlK-1 (kinase insert domain containing receptor/fetal liver kinase 1) [97]. This activates an MAPK pathway causing neovascular channel growth from the choroidal vasculature and extension into the space between the RPE and Bruch’s membrane thus activating the RPE to migrate into stroma of the CNV lesion [98, 99]. VEGF blockade has been shown to have a direct and rapid antivascular effect in tumors by deprivation of tumor vascular supply and inhibition of endothelial proliferation. Recently, VEGF has also been shown to target CNV in AMD [100]. The first anti-VEGF compound, pegaptanib, was approved by the FDA in 2004 and followed closely by approval of two other treatments, bevacizumab (Avastin) and ranibizumab (Lucentis). With monthly intravitreal injections of ranibizumab, growth of neovascular membranes is halted and there is prevention of severe vision loss in 90% of patients and improvement of visual acuity in 30–40% of patients [101–104].

### 5.2. Matrix metalloproteases, MMPs

The regulated turnover of extracellular matrix macromolecules is crucial to a variety of important biological processes. MMPs, a member of the class of proteases, degrade components of extracellular membranes [105]. MMPs, zinc-dependent endopeptidases, are expressed by activated macrophage foam cells and smooth muscle cells, and are important in the resorption of extracellular matrixes in both physiological and pathological processes. MMPs are secreted by macrophages as a proenzyme and once activated can completely degrade extracellular matrix components, such as elastin and collagen, including the structural backbone of the basement membrane, type IV collagen. Mostly this group of enzymes acts locally where they are expressed to aid in cell migration by clearing a path through the matrix, exposing cryptic sites on the cleaved proteins that promote cell binding and/or cell migration, promoting cell detachment so that a cell can move onward, or by releasing extracellular signal proteins that stimulate cell migration [105].

MMP-9, a specific MMP, is thought to degrade the fibrinous cap found on atherosclerotic plaques, destabilizing the plaque, and priming it for rupture [106]. Since AMD is associated with sustained chronic inflammation and loss of integrity of Bruch’s membrane, it has been hypothesized that MMPs may play a role in the pathogenesis of the disease [107]. MMP-9 and MMP-2, two subtypes of MMPs, have been identified in Bruch’s membrane in AMD eyes, and cell-culture studies have documented its role in the development of CNV [108–110]. A recent study found the first association between AMD and MMP-9 [108]. Significantly elevated plasma MMP-9 levels were reported in both wet and dry AMD patients as compared to age-matched controls. In addition, circulating plasma levels of MMP-9 were approximately three times higher in AMD patients than in control patients with no confounding illnesses. MMP transcriptional activity is regulated by genetic polymorphisms of the promotor region and carriers repeats of the MMP-9 promotor, numbering greater than or equal to 22, have a more than doubled risk of developing AMD [37]. Facilitating this MMP-9 expression may act as a factor in increasing vascular permeability of the vessels or in the neovascularization seen in exudative AMD.

### 5.3. Docosahexaenoic acid, DHA

Docosahexaenoic acid (DHA) is a major dietary omega-3 LCPUFA. It is also a major structural lipid of retinal photoreceptor outer segment membranes with the highest concentrations per unit weight found here. Omega-3 LCPUFA have the capacity to play roles in many processes of AMD, such as retinal neovascularization, inflammation of the retinal vasculature, and alterations in the retinal capillary structure and integrity [9]. DHA has been shown to promote survival, inhibit apoptosis of photoreceptors, possibly via signaling cascades, play a role in rhodopsin regeneration, and exert neural protection through an RPE-secreted neuroprotective mediator, NPD-1. Tissue DHA insufficiency can affect retinal signaling and is associated with alterations in retinal function [9]. It has also been documented that there exists an inverse relationship between dietary intake of the omega-3 LCPUFA and risk of developing AMD [111].

Despite the benefits of polyunsaturated fatty acids, humans lack the Δ15 and 12 desaturase enzymes to synthesize these compound de novo and are dependent on dietary sources. In addition, the biochemical nature of DHA and the proximity of these compounds to metabolically active ocular tissue and high oxygen tension of the choriocapillaries facilitate the formation of ROSs. ROSs may start an oxidative cascade altering the DHA and changing the composition of the cellular membrane and increasing the expression ofproinflammatory genes and cytokines, thereby damaging the retina [62, 63]. ROS are therefore extremely dangerous because they damage DHA, a necessary yet limited resource needed to keep retina healthy.

## 6. IMPORTANT MOLECULES INVOLVED IN PPAR’s POTENTIAL ROLE IN AMD

### 6.1. VEGF, PPAR*γ*, and their role in AMD

As previously discussed, VEGF has been shown to play a critical role in neovascularization via the MAPK kinase pathway, associated with the wet form of AMD [103, 104]. PPAR*γ* with expression localized to the RPE and choroidal endothelial cells of ocular tissue [53] may have an effect on endothelial cells and may have a direct antagonistic relationship with VEGF.

It has been demonstrated that vascular endothelial cells express PPAR-*γ* mRNA and protein [61, 112]. PPAR-*γ* ligands inhibit growth factor-induced proliferation of endothelial cells, increase plasminogen activator inhibitor-1 expression and suppress endothelin-1 secretion [113, 114], overall providing support to the theory that PPAR-*γ* plays an antagonistic role to that of VEGF [115]. More directly Murata and colleagues demonstrated that PPAR*γ* inhibits MAPK-dependent migration of smooth muscle and may act as a downstream inhibitor to VEGF. This group also showed that troglitazone and rosiglitazone, synthetic agonists of PPAR*γ*, inhibited the endothelial effects of VEGF in a dose-dependent manner. In vivo studies with the troglitazone demonstrated that intravitreal injections dramatically inhibited the percentage of lesions as well as leakage per lesion, making a strong case for therapeutic value of this drug [53].

### 6.2. Matrix metalloproteinase (MMP), PPAR*γ*, and their role in AMD

Ricote showed that PPAR*γ* inhibits the expression of MMP-9 in response to a naturally occurring ligand, prostaglandin D2 metabolite 15-deoxy-Δ^12,14^ prostaglandin J2 (15d-PGJ2), and synthetic PPAR*γ* ligands activated macrophages by antagonizing the activities of the transcription factors AP-1, STAT, and NF-*κ*B [52]. PPAR*γ* activators decrease MMP-9 expression in vascular smooth muscle [116] and treatment with PPAR agonist troglitazone has shown decreased atherosclerotic lesions in various animal models [107]. In addition PPAR*γ*-mediated suppression of NF-*κ*B activity may decrease proinflammatory cytokines in macrophages, including MMP-9 [117].

This intricate relationship demonstrates that PPAR*γ* downregulates MMP expression and inhibits MMP-9’s subsequent accumulation in Bruch’s membrane where it may play an integral role in the degradation of the extracellular matrix and be a stimulus for migration of the RPE into Bruch’s membrane, in this way contributing to the pathophysiology of AMD.

### 6.3. DHA, PPAR*γ*, and their role in AMD

DHA is a naturally occurring ligand to all subtypes of the PPAR family. It binds specific DNA motifs to modulate the activity of PPAR and RXR as transcription factors [9]. As being well known, PPARs play an important regulatory role in oxidative stress by inducing the transcription of antioxidant genes, such as glutamate cysteine ligase (GCL) and heme oxidase-1 (HO-1) [118]. These antioxidants then work through MAPK kinase pathways to curb ROS. A functional PPRE is located at the catalase gene promoter, a gene known to protect cells from the toxic effects of hydrogen peroxide (H_2_O_2_) by catalyzing its decomposition, indicating that catalase expression is directly regulated by PPAR*γ* [62]. To further test this relationship, catalase expression was analyzed in the striatum of rats subjected to intracranial bleeds with and without 15-dPGJ2 treatment. Treated rats showed 1.6-, 2.1-, and 1.7 fold higher levels of catalase mRNA expression compared to the saline controls at 1, 3, and 24 hours [63]. Girnun et al. found similar increases in catalase mRNA when using known PPAR agonists rosiglitazone and ciglitazone in rat brain microvascular endothelium cells, one of the cell types damaged during inflammatory responses induced by ROS generation [62].

In short, PPAR*γ* has a special role in counteracting the damaging effects of ROS generation by upregulating antioxidant genes and downregulating proinflammatory genes. By decreasing damage to LCPUFAs, such as DHA, there is preservation of the protective effects these essential molecules confer to the retina.Enhancing this ability of the RPE to protect itself from oxidative injury may provide a therapeutic opportunity to delay or hinder the development of AMD.

## 7. SUMMARY

**Figure 2:**
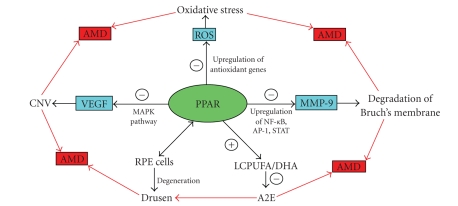
Schematic graph showing PPAR interactions with VEGF, ROS, MMP-9, LCPUFA, DHA, and RPE cells and their role in the development of AMD.

Though there is limited literature directly linking PPAR dysfunction with AMD pathology, there is evidence that PPARs may be involved in various mechanisms and pathways associated with this disease process. PPAR*γ* is localized to the neuroretina and RPE, the essential component to photoreceptor degeneration and vision loss. PPAR acts to inhibit inflammatory processes, which are linked to AMD. VEGF is a known driving factor for neovascularization, a main causal element of wet macular degeneration and PPARs directly inhibit VEGF function. High levels of MMP-9 have been detected in retinas afflicted with AMD. In turn, PPARs are known to decrease expression of MMP. PPARs play a direct role in upregulation of antioxidative enzymes, one of the many possible causes of macular pathology. PPARs bind various ligands including LCPUFAs and their metabolites, possibly shedding light on how PPARs interfere with NF*κ*B as one way in which omega-3 LCPUFAs are protective against AMD. It is evident that PPARs must play a certain role in the development of AMD. Figure 2 demonstrates the many ways that PPARs interact with processes closely related to progression of AMD. Future studies are warranted to better elucidate the pathogenic and therapeutic potentials of PPARs in AMD.
